# Physical Activity in Adolescents with and without Type 1 Diabetes during the New Zealand COVID-19 Pandemic Lockdown of 2020

**DOI:** 10.3390/ijerph18094475

**Published:** 2021-04-23

**Authors:** Deborah M. Telford, Dana M. Signal, Paul L. Hofman, Silmara Gusso

**Affiliations:** 1 Department of Exercise Sciences, Faculty of Science, University of Auckland, Auckland 1023, New Zealand; dtel007@aucklanduni.ac.nz; 2 Liggins Institute, University of Auckland, Auckland 1023, New Zealand; danez_01@hotmail.com (D.M.S.); p.hofman@auckland.ac.nz (P.L.H.); 3 Starship Children’s Health, Auckland 1023, New Zealand

**Keywords:** physical activity, Type 1 diabetes, adolescents, COVID-19

## Abstract

Physical activity (PA) is an important part of lifestyle management for adolescents with Type 1 diabetes (T1D). Opportunities for PA were reduced by COVID-19 restrictions. Therefore, the purpose of this cross-sectional study was to compare PA among adolescents with and without T1D during the first New Zealand (NZ) COVID-19 lockdown. PA levels of adolescents aged 11–18 years with T1D (*n* = 33) and healthy controls (*n* = 34) were assessed through self-reported and parent proxy-reported questionnaires. Overall, PA levels during lockdown were below recommended levels. PA levels did not differ between T1D and control participants (*p* = 0.212) nor between genders (*p* = 0.149). Younger adolescents tended to be more active than older adolescents (*p* = 0.079). PA level was negatively associated with BMI z-score (*r* = −0.29, *p* = 0.026) but was not associated with socioeconomic status (SES) or T1D-related parameters. In the T1D group, higher HbA1c was associated with lower school decile (*r* = −0.58, *p* < 0.001) and higher BMI z-score (*r* = 0.68, *p* < 0.001). Overall, young people were insufficiently active during lockdown, and some sub-groups were more affected than others by the restrictions. Pandemics are likely to be part of our future, and further studies are needed to understand their impact on the health and wellbeing of adolescents.

## 1. Introduction

Type 1 diabetes (T1D) is a chronic condition with elevated blood glucose levels caused by insulin deficiency from pancreatic ß-cell destruction [1]. T1D is a form of diabetes mellitus, which comprises several conditions characterised by high blood glucose levels. Diabetes mellitus affects around 463 million adults worldwide, amounting to 9.3% of the adult population [2]. In NZ, 260,000 adults are affected, or 7.7% of the adult population. Approximately 10% of these cases are T1D.

For adolescents with T1D, regular PA is a key component of lifestyle management promising many important health benefits including improved fitness, strength, body composition, weight management and self-esteem [3]. In addition, there is growing evidence that PA improves cardiovascular health in adolescents with T1D, which may help to reduce or delay diabetes complications [4,5]. Furthermore, physically active individuals with T1D have improved metabolic profiles compared with their sedentary counterparts with lower average glucose levels and fewer hyperglycaemic episodes [6].

It is recommended that adolescents with T1D engage in ≥60 min of moderate to vigorous PA (MVPA) per day [7,8] in line with guidelines for the general youth population [9]. However, engaging in PA can be challenging for individuals with T1D due to short-term disruption to glycaemic control and increased risk of hypoglycaemia [10]. Fear of hypoglycaemia is the most commonly reported barrier to exercise amongst young people with T1D [11]. Thus, young people with T1D must overcome these diabetes-related barriers in order to maintain an active lifestyle, in addition to the PA barriers commonly experienced by adolescents without T1D.

In a study of children and adolescents with T1D in Australia, 62% self-reported achieving more than 60 min a day of PA compared with 69% of children and adolescents in a nationwide survey [12]. However, comparative data is not available for young people with T1D in NZ. A number of studies worldwide have found that young people with T1D are typically less active and spend more time engaging in sedentary behaviours than their peers without the disease [13,14,15,16]. Furthermore, the majority of adolescents in the general population do not achieve the recommended levels of PA. An estimated 81% of students aged 11–17 years worldwide are insufficiently physically active [17], and in NZ, only 7% of young people aged 5–17 reported achieving PA guidelines in a nationwide survey [18]. Adolescence is a critical period for forming lifestyle habits, and sustained sports participation during adolescence is associated with higher levels of PA as an adult [19]. Therefore, it is critical from a public health perspective that all adolescents are encouraged to develop positive PA habits in order to improve long-term health outcomes. Considering the unique challenges faced by young people living with T1D, it is important to understand whether they are adhering to PA guidelines.

In January 2020, the WHO confirmed the presence of a novel coronavirus, SARS-CoV-2, causing a respiratory illness named COVID-19, and on 11 March 2020, COVID-19 was declared a pandemic [20]. As COVID-19 spread throughout the world, compulsory quarantine and nationwide lockdowns became necessary preventative measures to contain the pandemic. The WHO acknowledged the potential impact of COVID-19, issuing a series of recommendations which included supporting one another, creating and maintaining regular routines and engaging in healthy activities such as exercise [21].

The first case of COVID-19 in NZ was reported on 28 February 2020, and on 25 March 2020, NZ was placed under a nationwide lockdown. For 33 days, the country remained under lockdown at alert level 4 (maximum level), and everyone living in NZ was instructed to stay home, except when going out to obtain essential supplies or to exercise in the local area. All playgrounds and sports facilities were closed, and social distancing rules prohibited social contact with persons outside of the home or ‘bubble’. For a further 16 days, under alert level 3, social distancing rules remained in place and schools were closed to most students. Therefore, for a total of 7 weeks, most children and adolescents were schooled at home and did not have access to their usual PA routines. PA opportunities were limited to exercising in the local area or at home, either alone or with family living in the same house, whilst socially distancing from others. In the context of Auckland, NZ, this could include cycling or walking in local parks, beaches and reserves; performing home workouts or jumping on a trampoline at home, as trampolines are commonly owned by NZ families.

Thus, the COVID-19 pandemic resulted in compulsory lifestyle changes in NZ to prevent the spread of the disease. People with T1D are particularly vulnerable to the virus due to altered immune function [22,23], and the effects of pandemic-related stress may have implications for their overall health. Therefore, preserving immune function during pandemic lockdowns is an important consideration. Regular PA can strengthen the immune system, lowering the incidence and symptom intensity of viral infections [24]. Thus, maintenance of PA during pandemic lockdowns could have important benefits for population health [25].

There is a lack of data on PA levels of adolescents with T1D in NZ in relation to guidelines and in comparison to their healthy peers during normal times. It is also unknown how PA levels might vary with age, gender, SES and obesity within this clinical population. Furthermore, it is unknown what impact the pandemic had on PA levels of adolescents with T1D, who have increased vulnerability to COVID-19 and may therefore have higher stress levels and anxiety about the pandemic in comparison to their peers without T1D, factors which could influence their PA behaviour. Therefore, this study aimed to assess PA behaviours in adolescents with T1D in comparison to their peers without T1D in Auckland, NZ during the first nationwide lockdown of 2020. Secondary aims were to analyse overall PA levels of adolescents in relation to PA guidelines and to identify whether PA levels were associated with gender, age, socioeconomic status, BMI or diabetes-related parameters. This information may help to inform clinicians and health professionals on how pandemics affect young people with T1D and highlight potential areas of change to optimise healthcare management during such periods.

## 2. Materials and Methods

This was a cross-sectional study comparing the PA levels of adolescents in Auckland with and without T1D. Participants and their parents completed online questionnaires in May 2020 while NZ was emerging from full lockdown.

### 2.1. Recruitment and Participants

Adolescents with T1D aged 11–18 years living in the Auckland region were recruited from Starship Paediatric Diabetes Outpatient Clinics. Exclusions included those with T1D for <1 year, those with other severe illness (excluding coeliac disease), those with major behavioural problems or developmental delays which would prevent completion of assessments and those in need of an interpreter. A control group of 11–18-year-old adolescents without diabetes living in the Auckland region was recruited from the friends and family of the participants with T1D and from the wider community.

Prospective participants were informed about the study during their virtual or face-to-face diabetes clinic appointment. If they expressed interest, a telephone call was made to explain the study further and to enable participants to ask any questions. A consent form was signed by participants over 16 years of age and by a parent or caregiver for participants under 16 years of age. Participants aged under 16 years signed an assent form. In all cases, participants and parents were fully informed of the study details and provided with a copy of the study documents.

### 2.2. Procedures and Measures

Demographic data was collected from subjects by telephone or email. Due to COVID-19 restrictions, height and weight could not be obtained directly. For participants with T1D, the height and weight recorded at the latest diabetes clinic visit was obtained from electronic records. Control participants provided self-reported height and weight where they were able to do so. Instructions were provided to obtain height and weight without shoes and to measure height with feet together, standing up straight against the wall with heels touching the wall. Due to incomplete data being obtained from control participants, only those participants who could provide both height and weight were included in the analyses relating to BMI. BMI was calculated using the formula BMI = [weight (kg)]/[height (m)]^2^. Z-scores for height, weight and BMI were obtained using an online WHO anthropometric z-score calculator [26].

The New Zealand Deprivation Index 2018 (NZDep18) and school decile were used as indicators of socioeconomic status (SES). The NZDep18 is a measure of socioeconomic deprivation based on variables from the 2018 census, with scores ranging from 1–10, where 10 represents areas with the highest level of deprivation. Participant home address was matched to statistical area (SA1) using Statistics NZ online maps [27], and the corresponding NZDep18 score was recorded. School decile was recorded based on current school attended. School decile is calculated by the Ministry of Education and reflects the proportion of students within the school roll who come from low socioeconomic neighbourhoods. Deciles are denoted on a scale from 1–10, where lower decile represents lower income communities. Note that this is the inverse of the NZDep18 score, where a higher score represents greater deprivation.

Participants with T1D provided their current insulin regime, method of blood glucose monitoring, date of diagnosis with diabetes (month, year) and details of any severe hypoglycaemic episodes or hospital admissions with DKA in the past 3 months. This information was also verified by medical records.

The Physical Activity Questionnaire for Older Children (PAQ-C) [28] and the Physical Activity Questionnaire for Adolescents (PAQ-A) [29] were used to assess self-reported PA levels in 11–12 year-olds and 13–18 year-olds, respectively, and are collectively referred to as the PAQ herein. The Children’s Physical Activity Questionnaire (CPAQ) was used to obtain parents’ assessments of their children’s PA [30]. All questionnaires were delivered via Qualtrics (Qualtrics, Provo, UT, USA, 2018).

### 2.3. Statistical Analysis

IBM SPSS Statistics version 26 software (Armonk, NY, USA) was used to perform all statistical analyses. Statistical significance is defined as *p* < 0.05, and results are expressed in the form of mean ± standard deviation, unless stated otherwise. For significant results, effect size is reported using Cohen’s *d* or η^2^. It was not feasible to perform a priori power analysis to determine sample size, as recruitment and data collection were limited by the brief duration of the lockdown period. Post hoc power analysis using G*Power indicated that the sample size was sufficient to detect a large effect size (*f* = 0.4) in the 3-way analysis of variance (ANOVA) with power of 0.90, or a moderate effect size *(f* = 0.25) with a power of 0.52 with alpha = 0.05

Baseline characteristics were compared using the student’s *t*-test for numerical variables and the *X*^2^-test or Fisher’s exact test for categorical variables. Multiple regression analysis was performed to explore the effects of (i) having T1D and (ii) SES (school decile) on BMI z-score because school decile differed significantly between the T1D and control groups. For participants with T1D, to investigate the associations between HbA1c and other variables (diabetes duration, school decile, NZDep18, BMI z-score), correlation analysis was performed. For simplicity, the 10-point scales for school decile and NZdep18 were treated as continuous variables.

To assess PA in relation to recommended levels of MVPA, the PAQ cut-off score of 2.75 derived by Benitez-Porres et al. [31] was adopted. To test whether T1D, age and gender had an effect on PA levels, and to assess whether these three variables interacted with one another, PAQ scores were analysed using 3-way ANOVA with 3 between-subjects factors: group (T1D, CON); age (11–14 years, 15–18 years) and gender (M, F). The decision to dichotomise age into two groups (<15 years old, ≥15 years old) was based on evidence that activity levels tend to decrease from around 15 years of age onwards in the NZ adolescent population [32] and worldwide [17], suggesting that these two age groups might have distinct PA profiles. Correlation analyses were performed to explore the associations between PAQ score and other continuous variables (BMI z-score, school decile and NZDep18).

To analyse the effects of diabetes parameters on activity levels, the following analyses was performed on T1D participants only: (i) partial correlation analysis between recent HbA1c and PAQ score, controlling for age; (ii) partial correlation analysis between diabetes duration and PAQ score, controlling for age; and (iii) one-way ANOVA with total PAQ score as the dependent variable and method of insulin delivery as a between-subjects factor with 3 levels (multiple daily injections [MDI], twice-daily injections [BID], pump).

To establish the reliability of self-reported PAQ scores, correlation analysis was performed between parent proxy-reported CPAQ total active hours and self-reported PAQ score. CPAQ total active hours was obtained by calculating the total hours reported across the three domains (sport, leisure and school) and subtracting time recorded for the items “playing with pets” and “household chores”, since these were deemed to be low-intensity activities. An independent samples *t*-test was performed to compare the parent-reported mean active hours of T1D and CON participants.

## 3. Results

### 3.1. Demographics and Participant Characteristics

Of the 310 individuals aged 11–18 in Auckland T1D clinics, 110 were invited to participate who met the study criteria and attended clinics during the recruitment period. Of these 110 individuals, 38 (35%) expressed interest and provided consent. Four did not complete the online questionnaire for unknown reasons and one was excluded due to being diagnosed with a different type of diabetes. Therefore, 33 participants with T1D were included in the analyses. Thirty-eight healthy controls were contacted, and 34 consented to participate, including 9 siblings of T1D participants. All 34 control participants completed the questionnaires and were included in the analyses. The demographics of the study participants are shown in Table 1. The groups were similar in age, gender and ethnicity, but SES was higher in the control group than in the group with T1D. Overall, 84% of participants were of European heritage, 7% were of Māori ethnicity and 6% were of Pacific ethnicity.

The physical characteristics of the study participants are described in Table 2. Complete height, weight and BMI data could not be obtained for 6 control participants due to the requirement to self-measure under COVID-19 restrictions.

Overall, 45% of participants with T1D were classified as overweight or obese based on WHO BMI z-score cut-off points of >1 and >2 respectively, with 12% in the obese category and 33% overweight. There were no controls classified as obese, while 21% of controls were classified as overweight. As shown in Table 2, average BMI z-score was significantly higher for the T1D group (*p* = 0.006, d = 0.73). To establish whether this difference was due to differing SES between the two groups, a multiple linear regression was performed to predict BMI z-score based on school decile and group (T1D, CON). The resulting equation indicated that a decrease in school decile of 1 strongly predicted an increase in BMI z-score of 0.14 (*p* < 0.001), and having T1D tended to predict an increase in BMI z-score of 0.50 (*p* = 0.058). The overall model fit was *R*^2^ = 0.244.

As shown in Table 2, average HbA1c was 72 mmol/mol (8.7%) (range 48–130 mmol/mol (6.5–14%)) based on the most recent measurement, which was high in relation to the internationally recommended target (≤53 mmol/mol (≤7%)) for children and adolescents with T1D [33]. HbA1c was negatively correlated with school decile (*r* = −0.58, *p* < 0.001) and had a modest, non-significant, positive correlation with NZDep18 (*r* = 0.33, *p* = 0.065), indicating that those living and attending schools in more socially deprived areas tended to have poorer glycaemic control. There was a moderate-to-strong positive correlation between HbA1c and BMI z-score (*r* = 0.68, *p* < 0.001), indicating that being overweight/obesity was associated with poorer glycaemic control. There was no clear association between HbA1c and diabetes duration (*r* = −0.17, *p* = 0.348). Hence, poorer glycaemic control was associated with higher BMI and with greater socioeconomic deprivation but not with diabetes duration.

### 3.2. Self-Reported Physical Activity

Overall, the participants achieved a low-to-moderate level of PA during the first NZ COVID-19 lockdown, indicated by an average PAQ score of 2.36. As shown in Table 3, there was no difference in activity levels between participants with and without diabetes, nor between males and females. There was a moderate difference in mean PAQ score between younger and older adolescents that was approaching significance (*p* = 0.079), indicating that the younger group tended to be more active than the older group. No interactions were observed in PAQ score between age, gender and group, indicating that the effects of these factors on PA were independent of each other.

PAQ score was negatively associated with BMI z-score (*r* = −0.29, *p* = 0.026), indicating that those with higher BMI tended to be less active, with BMI explaining approximately 7% of PAQ score variance. PAQ score was unrelated to either school decile (*r* = 0.10, *p* = 0.431) or NZDep18 score (*r* = −0.11, *p* = 0.377), indicating a similar level of activity regardless of level of social deprivation.

Within the T1D group, controlling for age, there was no clear relationship between PAQ score and HbA1c level (*r* = −0.17, *p* = 0.356), nor between PAQ score and diabetes duration (*r* = 0.177, *p* = 0.334). Average PAQ scores did not differ significantly between methods of insulin delivery: BID: 1.94 ± 0.93; MDI: 2.46 ± 0.60; pump: 2.30 ± 0.59, *F*(2,29) = 1.14, *p* = 0.333.

### 3.3. Parent Proxy-Reported Physical Activity

Parent proxy-reported CPAQ questionnaires were received for 57 participants (T1D: *n* = 29, CON: *n* = 28). Parent proxy-reported total active time was moderately correlated with self-reported PAQ score (*r* = 0.60, *p* < 0.001), indicating a substantial degree of agreement between these two measures. Overall, according to parents, participants spent an average of 8.2 ± 6.3 h/week in PA across all domains (sport, leisure and school). There was no significant difference in parent-reported active hours between the control group (9.2 ± 6.6) and T1D group (7.3 ± 6.0), *p* = 0.243.

Within each domain, time was reported across a range of activities. The most popular sports and leisure activities were walking, trampolining, bike-riding, dancing, running and gymnastics. Those who participated in dancing, gymnastics, martial arts and basketball spent the most time practicing their sport (approximately 30–40 min per day). Home-based activities including dancing, gymnastics and aerobics were more popular with girls, with 49% participating in one or more of these activities, compared with 15% of boys.

## 4. Discussion

This study offers a unique insight into the PA levels of adolescents with T1D and their healthy peers during the first COVID-19 lockdown of 2020 in NZ, when sport and leisure activities were restricted. The main aim was to compare PA between adolescents with T1D and healthy controls during lockdown. Our results indicate that while PA levels did not differ between T1D and control adolescents during this period, both groups were insufficiently active.

There are currently no other cross-sectional data comparing lockdown PA levels between adolescents with T1D and healthy controls. Pre-pandemic data, however, indicates that adolescents with T1D tend to be less active than their peers [14,15,16], and studies of individuals with T1D during lockdown without a control group indicate that their PA levels were lower than usual [34,35,36]. While it was encouraging to find that those with T1D exercised at a similar level to their peers without diabetes during the lockdown period in our study, the average PAQ score of 2.36 for all participants was well below the estimated cut-off of 2.75 for meeting minimum recommended levels of MVPA [31], with approximately two-thirds failing to meet this threshold. Furthermore, total active time reported in the CPAQ (8.7 h/week) was lower than the average weekly PA for young people aged 12–17 (11 h/week) reported in the nationwide Active NZ Survey 2019 [18]. While caution must be taken comparing these data sources due to methodological and demographic differences, the evidence suggests that PA levels during lockdown were lower than usual for adolescents with and without T1D.

Global research indicates a reduction in PA and increase in sedentary behaviour during pandemic-related movement restrictions amongst young people in China [37], Croatia [38], Ireland [39], Spain [40], the US [41], Saudi Arabia [42] and Australia [43]. A similar pattern of reduced activity worldwide has been reported in adults during the pandemic [44], with few exceptions [45]. Two studies have used the PAQ-A to assess PA in adolescents. Elnaggar et al. [42] assessed PA levels in 63 healthy adolescents aged 14–18 years in Saudi Arabia, reporting an average PAQ-A score of 2.77 during movement restrictions compared with 3.05 at baseline. Similarly, Sekulic et al. [38] reported an average PAQ-A score of 2.67 after initiation of social distancing, compared with 2.99 at baseline amongst 388 adolescents aged 15–17 years in Croatia. The reason for the lower average score of 2.39 for our NZ cohort compared with 2.67–2.77 in these two studies is not clear. However, differences could arise from variations in the local restrictions in place at the times of the surveys, such as lockdown versus social distancing only, or characteristics of the study participants, such as age, gender and health status. Thus, it seems levels of PA during lockdown reported in our study were lower than usual, in line with existing worldwide research.

Amongst the participants with T1D, our results indicated that there was no relationship between PA levels and glycaemic control. The results indicated that having poor glycaemic control was not a consistent barrier to PA during lockdown, and that some participants with very high HbA1c levels were able to stay physically active despite this. Research assessing the relationship between glycaemic control and PA in adolescents with T1D during the pandemic is very limited. One small study of 13 children and adolescents with well-controlled T1D in Italy noted that maintaining PA at home during lockdown was associated with improved glycaemic control [46], and pre-pandemic research indicated that higher HbA1c was associated with greater barriers to PA in children and adolescents with T1D [11]. However, while some studies have found HbA1c levels to be inversely associated with PA levels in young people with T1D [14], others have failed to detect any correlation [47].

Higher HbA1c was associated with lower SES and higher BMI. As there was no relationship between SES and activity level, the tendency for individuals from more socially deprived areas to have poorer glycaemic control may be influenced by lifestyle factors other than PA behaviours such as dietary habits or poor treatment compliance. The positive association between BMI and HbA1c also suggests that dietary factors may be contributing to poorer glycaemic control. In order to reach those who are most at risk, future studies looking into PA and glycaemic control in adolescents with T1D should examine dietary habits, particularly amongst those living in areas of high deprivation where economic factors may be contributing to poor food choices.

Our results indicated that activity levels were similar for male and female adolescents during lockdown. Based on pre-pandemic data, male children and adolescents in NZ tend to be more active than their female counterparts, typically engaging in 90 min more activity per week [32]. However, emerging evidence suggests that boys tended to decrease their PA to a greater extent than girls during the pandemic, resulting in a smaller difference in PA between genders during this period [37,38,42]. A possible explanation for this is differences in activity choices, with boys preferring competitive team sports not available during lockdown and girls being more inclined to perform home workouts such as aerobics, dance or gymnastics [37,38]. Our data supports this, with 49% of girls participating in home workouts compared with only 15% of boys. This suggests that initiatives to keep people active during the NZ lockdown such as televised workouts were likely to have appealed more to girls than boys. Therefore, it is important during future lockdowns to devise novel ways to encourage boys to stay active.

The PAQ results indicated that younger adolescents tended to be more active than older adolescents. While pandemic data for PA by age group is not available, based on pre-pandemic data, average PA decreases steadily throughout adolescence [48], and in NZ, average active time decreases from 12.0 h/week in 12–14-year-olds to 9.6 h/week in 15–17-year-olds [32]. A factor contributing to this decline is reduced participation in school physical education, which is not compulsory in NZ schools beyond year 10 (age 14–15 years). Our results indicate that similar age-related trends in PA were present during lockdown as have been reported in the pre-pandemic literature. Nevertheless, it is possible that all ages were not equally affected by the restrictions. Since participation in competitive sports and activities is highest between the ages of 8 and 14, the lack of organised sporting activities may have had a greater impact on PA in younger adolescents [17]. In addition, younger adolescents may be less inclined or able to leave home to exercise independently compared with older adolescents. Therefore, while more evidence is needed to confirm the precise impact of lockdowns on different age groups, it is important to consider age-related factors when promoting PA during future lockdowns.

BMI was negatively associated with activity levels during lockdown. Studies in other areas have also produced evidence of a relationship between BMI and PA during the pandemic. A study of Irish adolescents found that those who were overweight or obese were approximately twice as likely to report performing less PA than usual during lockdown compared with those who were classified as normal weight, although overall lockdown activity levels were unrelated to BMI status [39]. In contrast, Elnaggar et al. [42] found no association between BMI and reported change in PA amongst Saudi Arabian adolescents during the pandemic but reported a significant inverse correlation between BMI and PA, both at baseline and during lockdown, in males only. Thus, while BMI appears to have influenced PA levels during the pandemic, the results are varied and inconsistent. The inconsistency of these results may stem from cultural or demographic differences in the populations being studied or differences in the types of exercise being performed. Our results, however, must be interpreted with caution, as the BMI data for the control group was incomplete and was indirectly obtained through self-measurement at home. Nonetheless, our finding that overall activity levels during the lockdown period in NZ tended to be lower in those with higher BMI gives grounds for further investigation to understand the relationship more fully. This finding also highlights the need to ensure that PA opportunities are created that are appropriate and appealing for all sectors of society during lockdown situations.

The overall low levels of PA amongst young people during pandemic-related movement restrictions reported in this study and elsewhere are concerning. Lockdowns are likely to be part of the future for the young people of today. Therefore, it is important that this generation of youth are encouraged to develop strong PA habits which enable them to continue to be active during challenging times such as pandemic lockdowns as they grow into adulthood. This is particularly important as an estimated 27.5% of adults are insufficiently physically active during normal times [49].

### Strengths and Limitations

To our knowledge, this was the first study to compare PA levels between adolescents with T1D and healthy controls during the COVID-19 pandemic and the first study to explore PA behaviour in adolescents during the COVID-19 lockdown in NZ. Use of internationally validated questionnaires and inclusion of parent proxy-reported data in addition to self-reported PA ensured good reliability and validity of the data. In addition, the investigation of a wide range of potential correlates of PA including anthropometric, demographic and T1D disease-related parameters provided a comprehensive examination of activity levels of young people during this period of pandemic-related movement restrictions in NZ.

We do, however, need to interpret our results with caution, as the study had a few limitations. Firstly, sample size was limited by the requirement to terminate recruitment and data collection at the end of the lockdown period and by the number of clinical appointments scheduled for this period. Possible relationships between PA and ethnicity were not explored due to the low number of non-European participants. Self-reported PA levels were a further limitation due to the risk of individuals reporting higher levels of activity than were actually performed. However, this risk was mitigated by use of parent-reporting to corroborate self-reported activity levels. Additionally, adherence to the guideline of <2 h recreational screen time was not assessed. Finally, we were unable to obtain pre-pandemic PA levels for our participants because the risk of recall bias was considered too high to collect this data retrospectively. Therefore, our discussion of the effects of the pandemic on PA levels relies on data collected prior to the pandemic from large samples of NZ adolescents within the general population.

## 5. Conclusions

There was no difference in physical activity levels between adolescents with and without T1D. However, our results highlight that young people in NZ with and without T1D were not sufficiently active during lockdown, consistent with findings in other countries. Thus, it is important that strategies and guidelines are developed to encourage young people to stay active during situations when movement restrictions are in place. These strategies should be developed to target distinct sub-groups, taking into account age, gender, social deprivation and health status rather than adopting a one-size-fits-all approach.

## Figures and Tables

**Table 1:** Participant demographics.

	T1D	Control	*p*-Value
Number of participants, *n*	33	34	
Female, *n* (%)	19 (58%)	25 (74%)	0.170 ^1^
Age (years)	14.1 ± 1.6	14.6 ± 1.9	0.267 ^2^
SES (NZDep18)	4.7 ± 3.1	3.5 ± 2.0	0.063 ^2^
SES (School decile)	6.2 ± 3.2	7.6 ± 2.2	0.037 ^2,^*
Ethnicity			
European	26	30	0.747 ^3^
Māori	3	2	
Pacific	3	1	
Other	1	1	

^1^*X*^2^ test; ^2^ Student’s *t-*test; ^3^ Fisher’s exact test; * *p* < 0.05.

**Table 2:** Participant characteristics.

	T1D	Control	*p*-Value ^1^
Number of participants, *n*	33	28 ^2^	
Weight (kg)	59.5 ± 12.1	55.6 ± 13.9	
Weight *z*-score	0.78 ± 1.02	0.33 ± 0.97	0.079
Height (cm)	163 ± 9	164 ± 10	
Height *z*-score	0.42 ± 0.93	0.51 ± 0.80	0.670
BMI (kg/m^2^)	22.3 ± 3.5	19.9 ± 3.2	
BMI *z*-score	0.86 ± 0.98	0.13 ± 1.00	0.006 **
Diabetes duration (years)	6.2 ± 3.3	*n*/a	
HbA1c (most recent) (mmol/mol)	72 ± 22	*n*/a	
HbA1c (1 year average) (mmol/mol)	71 ± 21	*n*/a	

^1^ Student’s *t-*test; ^2^ Six control participants were unable to provide height and weight data; ** *p* < 0.01.

**Table 3:** Results of 3-way ANOVA comparing PAQ scores by group, age-group and gender.

	*n*	PAQ Score Mean ± SD	*F*	*p*-Value	Cohen’s *d*
All	67	2.36 ± 0.71			
**Group**					
T1D	33	2.23 ± 0.68	1.59	0.212	0.17
Control	34	2.48 ± 0.71			
**Age-group**					
11–14 years	45	2.52 ± 0.71	3.21	0.079	0.70
15–18 years	22	2.03 ± 0.59			
**Gender**					
Male	23	2.28 ± 0.74	2.14	0.149	0.33
Female	44	2.51 ± 0.61			
**Interactions**					
Group*Age-group			0.983	0.326	
Group*Gender			0.116	0.735	
Gender*Age-group			0.658	0.420	
Group*Gender*Age-group			0.029	0.864	

## Data Availability

The data presented in this study are available on request from the corresponding author. The data are not publicly available due to privacy reasons.
